# Supplementary material for: Are carbon emissions trading and green financial instruments synergistic? -Comprehensive quantitative research based on content analysis
Source: PLoS One. 2024 Mar 7;19(3):e0298601. doi: 10.1371/journal.pone.0298601 (PMC10919601; doi:10.1371/journal.pone.0298601)
Supplement: S1 Data — (ZIP) [file pone.0298601.s001.zip › Data/地级市Z_F工作报告.zip!/╜¡╬≈/╕╙╓▌╩╨/└┤╫╘╣1⁄2╓┌║┼í╛┬φ┐╦╩2╛▌═°í┐_2003.docx]

各位代表：

现在，我代表市人民政府，向大会作政府工作报告，请予审议，并请政协各位委员和列席会议同志提出意见。

一、2002年的工作回顾

过去的一年，在中共赣州市委的正确领导下，我市经济发展取得了较好成绩，速度加快，投入增加，质量提高，后劲增强，完成了市一届人大四次会议确定的各项任务。

国民经济持续增长，运行质量不断提高

实现国内生产总值308.5亿元，增长10.6%，比上年提高2.6个百分点；实现财政总收入26亿元，增长10.5%，地方一般预算收入17.3亿元，增长5.1%。农村经济稳定发展。实现农业总产值146亿元，增长3.1%；农业结构继续优化，以脐橙为主的果业、以草食动物为主的畜牧业、以外销为主的蔬菜业和园艺花卉业取得新进展；农业产业化进程加快，年产值或销售收入500万元以上的龙头企业61个，农产品商品率达69%。工业经济较快增长。规模以上工业企业实现工业增加值24.5亿元，实现税金6.38亿元，分别增长18%和11%；扶优扶强成效明显，全市28户重点企业产销两旺，效益大幅上升；工业园区建设进展较快，市、县两级20个工业园共完成“三通一平”面积4.5万亩，已入园企业526个。第三产业发展良好。实现增加值116亿元，增长12.7%;商贸流通和旅游业迅速发展，城乡市场繁荣活跃，物价平稳，社会消费品零售总额109.5亿元，增长10.1%，居民消费价格总指数为100.7%。

改革开放力度加大，经济发展活力增强

企业改革步伐加快。全市国有企业改制面达88.6%，市直单位所属企业改革攻坚取得明显成效，通过多种形式的改制，一批企业焕发了生机，形成了新的增长点。农村改革不断深化。农村税费改革稳步推进，农民减负率达到48.4%。其它配套改革全面推进。政府机构改革进展顺利；财政、投融资、公路和流通体制改革继续深化；科技、文化、教育、卫生、保险、水利、林业等体制改革有序推进。对外开放进一步扩大。实际利用外资1.6亿美元，增长50%；实际引进内资71.95亿元，增长132%。非公有制经济快速发展。全年个私企业新增注册资本26.1亿元，增长94.7%；新增注册资本100万元以上私营企业408户，增长46.8%；实现工商税收7.7亿元，增长22.2%。

重点工程进展顺利，基础设施和城镇建设步伐加快

全年完成全社会固定资产投资109亿元，增长50%，其中重点工程投资22.63亿元。赣定高速公路、赣龙铁路等一批重点工程进展顺利；公路建设完成投资8亿多元，国省道改造500公里，竣工230公里，建成县乡水泥（油）路240公里；城乡电网改造基本完成；技改投资增幅提高，新开工项目增多；全市完成市政基础设施投资11.1亿元，新增城市建成区面积21.4平方公里，新增、改建城市道路面积183万平方米；村镇建设完成投资16.71亿元；市政中心等一批重点工程建成投入使用。

各项社会事业协调发展，人民生活继续改善

科技对经济增长的贡献率进一步提高；教育事业有较大发展，农村义务教育得到巩固；城乡医疗卫生状况得到改善；全民健身运动蓬勃开展；文化艺术事业健康繁荣；全市人口自然增长率9.54‰，计划生育率87.41%，较好地完成了人口计划和计生工作目标任务；环保工作力度加大，环境质量明显改善；劳动和社会保障事业有新进展，企业离退休人员养老金按时足额发放，国有企业下岗职工基本生活得到保障，失业保险参保人数增加，城市居民最低生活保障逐步走向法制化、制度化；新阶段扶贫开发工作全面展开；创建全国优秀旅游城市工作深入扎实；土管、技术监督、广电、民政、审计、统计、监察、人事、人防、精神文明建设、民族宗教、外事侨务、档案、保密、地震、气象、地方志、老龄等各项事业和国防、民兵、预备役部队建设等取得了新成绩；金融、通讯、海关、出入境检验检疫等服务行业为全市经济建设发挥了积极作用；人民生活水平继续提高，城市居民可支配收入6175元，增长16.4%，农民人均纯收入2190元，增加77元；社会稳定和安全生产工作常抓不懈，确保了全市无重特大安全事故，全市治安秩序良好，社会政治稳定；信访工作有了加强；各级政府接受人大、政协、民主党派和人民群众监督的自觉性不断提高，民主法制建设进一步强化；廉政建设和反腐败斗争力度加大，取得较好成效。

过去的一年，我们取得的成绩来之不易，这是全市人民锐意进取、团结奋斗的结果。在此，我代表市人民政府向全市广大干部群众，向驻市解放军和武警官兵，向所有关心和支持我市建设的国内外朋友以及社会各界人士，表示崇高的敬意和衷心的感谢！

各位代表，回顾一年来的工作，最根本的一条，就是我们始终坚持把发展作为执政兴国的第一要务。一年来的实践告诉我们：加快发展步伐，重在解放思想，扬优乘势。必须把解放思想贯穿经济和社会发展的始终，从赣州实际出发，在深化改革和扩大开放中，不断地解放思想，与时俱进，形成改革开放的强大动力，把发展推向新阶段。抢抓发展机遇，重在突出重点，力创特色。必须紧扣结构调整这一主线，把握事关全局的重点，按照“希望在山、在非、在特”的要求，加大主攻工业的力度，提高工业在国民经济中的份额。加快农业产业化步伐，放手发展非公有制经济，力创区域经济的产业特色。破解发展难题，重在知难而进，勇于创新。必须敢于和善于破解资金紧缺的难题，消除畏难情绪，创新招商引资、启动民资、向上争资、银行贷资、社会融资的思路，不断加大对经济和社会发展的投入。实现发展目标，重在群策群力，狠抓落实。必须形成团结兴市的共识和合力，正确处理改革、发展和稳定的关系，坚持稳定压倒一切。充分调动广大干部群众的积极性和创造性，把各项工作措施落到实处。

在肯定成绩的同时，我们也清醒地看到发展中还存在不少困难和问题。主要表现在：经济总量、发展速度、人均水平与“站前列、争一流”的要求还不相适应。我市GDP增速仅与全省平均水平持平，且经济总量小，人均水平低，发展差距仍在拉大。对外开放、招商引资水平与赣州处于江西省改革开放前沿阵地还不相适应。开放水平还不够高，发展环境还有不尽人意的地方，招商引资的发展速度不够快，引进的大企业、技术含量高的项目不多。经济结构与赣州的资源优势还不相适应。我市三大产业中二产比例明显偏低，比全国、全省分别低22和6个百分点。具有明显优势的资源开发利用不够，尚未形成规模大、效益好、竞争力强的产业。经济发展水平与改善人民生活的要求还不相适应。部分城市居民和企业职工生活比较困难，农民增收缓慢，有的地方拖欠职工工资，就业和社会保障压力加大。政府工作与新形势、新要求、新任务还不相适应。政府职能转变滞后于市场经济发展，行风不正和消极腐败现象依然存在，公务员的素质有待进一步提高。这些问题需要我们高度重视，在深化改革和加快发展中切实加以解决。

二、2003年政府工作的主要目标和任务

2003年政府工作的总体要求是：以邓小平理论和“三个代表”重要思想为指针，全面贯彻落实党的十六大和中央经济工作会议精神，进一步深化改革，扩大开放，以招商引资为突破口，把扩张以工业为核心的经济总量作为重点，加大投入，做强工业，做优农业，做美城市，加快发展速度，改善发展环境，提高发展质量，改进政府工作，为全面建设小康社会开好头、起好步。

经济和社会发展的主要预期目标是：国内生产总值增长11%；全社会固定资产投资增长30%；实际利用“三外”资金增长20%；社会消费品零售总额增长9.5%；财政总收入增长11%；城市居民可支配收入和农民人均纯收入分别增长9%和4.5%；城镇登记失业率和人口自然增长率分别控制在4.5%、9‰以内。

主要工作任务是：

（一）调整和优化经济结构，争创产业发展的新优势。

做大做强工业经济。坚持扩张总量与提高质量并举，培育重点骨干企业与壮大优势特色产业并重，改造传统产业与发展高新技术产业并进，走新型工业化道路。以更大的精力抓工业发展，以更新的思路谋划工业发展，以更实的措施促进工业发展，努力实现高于GDP增速8个百分点以上的工业发展目标。加强支柱产业建设。围绕提高企业的核心竞争力，加快技术创新步伐，积极运用高新技术改造传统产业，发展壮大新材料、食品和生物制药三大支柱产业。尤其是抓好以稀土和钨为主的新材料工业，促成与国内外大企业、大集团的合作，促进新发现的资源在更合理的机制下获得开发利用。集中力量抓好40户重点企业，实现利税总额增长20%以上，销售收入过亿元、利税总额超千万元的企业分别达到18户的目标。培植壮大优势名牌产品，提高工业产品的市场占有率。抓紧建设60万吨以上旋窑水泥、有色金属矿产开发和深加工等一批项目。同时，面向沿海发达地区，跟进接替转移产业，发展劳动密集型企业，增强工业发展后劲，壮大工业总量。

推进农业产业化进程。按照依法、自愿、有偿的原则，推进土地经营权的流转，进一步制定和落实好鼓励政策及帮扶措施，通过租地经营、股份制等形式，引导种养大户、生产能手、工商企业和外来投资者成片承包土地、山林和果园，提高农业生产的规模化和集约化水平。加快主导产业发展，集中力量抓好果业、商品蔬菜、草食畜禽和特种水产、园艺花卉等主导产业，培育一批有地方特色的名牌产品。大力发展脐橙产业，坚持一手抓开发，一手抓提高，夯实赣州建成为世界著名脐橙主产区的基础。加大科教兴农力度，抓好农业示范基地,加快农产品标准化体系和农业社会化服务体系建设。发展壮大龙头企业，鼓励社会资本和外资创办及嫁接改造各种龙头企业，努力打造一批农产品加工、销售企业。抓好农产品市场培育，引导农民走向市场，搞活流通，加快培育农民经纪人队伍、行业协会、专业合作社等新经济组织。进一步增加农业投入；加强水利基础设施建设；继续搞好新阶段的扶贫开发；建设以“猪沼果”为重点的生态农业，促进农业和农村经济全面发展，增加农民收入。

加快第三产业发展。把旅游业作为重要支柱产业来培育，调动社会各方面的力量投资旅游业，加大旅游资源开发力度，建成一批主题突出、特色鲜明的旅游精品，争取创建全国优秀旅游城市一举成功。完善房地产二、三级市场的各项政策，降低交易成本，扩大住房信贷，激活房地产市场。大力培育劳动力、资本、技术、产权等要素市场。积极探索现代物流业和会展业的发展路子。支持金融体制改革，发展现代金融服务业。扶持和发展社区服务业，推进企业和单位服务业社会化进程，逐步形成适应发展要求的现代服务业体系。

（二）深入推进市场取向改革，实现体制创新的新突破

在国有企业改革上求突破。加快国有资本退出步伐。以完善、深化、扩展为着力点，通过改革淘汰破产一批、放开搞活一批、壮大提高一批，增强企业活力，促进工业发展。推进林业、农业、水利、交通、建设等系统的企业改革。围绕发展壮大优强企业，政策上扶持，环境上改善，制度上激励，帮助南方稀土高科、赣南海欣等企业作好上市准备。按照国家所有、分级管理的原则，构建政企、政资、政事三分开，管人、管事、管资产三结合的新型国有资产管理体系。

在优化所有制结构上求突破。大力发展非公有制经济，鼓励非公有制企业大胆进入法律法规未予限制的所有经营领域，不限发展比例、速度、规模和方式；鼓励非公有制企业采取资本注入、技术转让等方式，参与国有企业的资产重组和结构调整；鼓励非公有制企业吸纳社会资本，不断提高产权混合度；鼓励金融机构开拓新的金融业务，加大对非公有制经济发展的信贷支持。引导和支持非公有制企业搞好产品经营和资本经营，走公司化、集团化、国际化的道路。强化对民营业主和个体劳动者的教育引导和监督管理，帮助他们提高素质，逐步形成一批实力强、素质高的企业家队伍。进一步优化非公有制经济发展的舆论环境，在全社会形成鼓励创业者、厚爱纳税人的良好氛围。

在深化各项配套改革上求突破。加强社会保障体系建设，坚持不懈地巩固“两个确保”，做好“三条保障线”衔接工作。扩大社会保险覆盖面，切实加强养老、失业保险的扩面征缴工作，努力拓宽保费来源。稳步推进全市医疗保险体制改革。落实和完善再就业的各项政策，广开就业门路，千方百计做好下岗失业人员特别是“4050”人员的就业和再就业工作，切实关心和帮助困难群众的生活。继续深化财政体制改革。建立和完善公共财政体制，深化支出管理改革，进一步推行部门预算，加强预算外资金管理，完善“收支两条线”制度，全面实行综合预算，规范财政供给范围。全面推进政府采购制度，继续完善会计委派制度，强化财政监督职能，严格依法治税。努力实现保工资、保法定支出、保稳定的目标，确保全年财政收支平衡。推进事业单位改革。巩固农村税费改革成果，切实减轻农民负担。

（三）继续实施大开放主战略，开创对外开放的新局面

把优化环境作为扩大开放、加快发展的重中之重来抓。营造优质高效的服务环境。建立综合审批绿色通道，把需要审批的事项全部进入行政服务中心，实行“一站式办公、一条龙服务”。推行首问负责制、服务承诺制和限时办结制，加快推进“电子政务”进程，完善配套服务，切实降低企业发展的综合成本；营造公正严明的法制环境。加大环境整治力度，严格规范行政执法行为，严厉查处“三乱”，严禁暗箱操作，实行阳光政务；营造公平竞争的市场环境。继续整顿和规范市场经济秩序，打击制假售假、偷税骗税、经济欺诈、逃废债务等不法行为，保护投资者的合法权益；营造诚实守信的信用环境。强化全民的信用意识，加强社会信用体系建设，倡导诚信守法、崇尚文明的社会风气，树立起良好的政府信誉、企业信誉和社会信誉，着力打造“信用赣州”，把赣州建设成为亲商、安商、富商、投资环境最好的地方。

把招商引资作为扩大开放、加快发展的突破口来抓。加大招商引资力度，做到思想更加解放，思路更加开阔，措施更加过硬，工作更加扎实。瞄准国内外大公司、大财团，在项目推介、综合服务等方面采取措施，尽快引进一批大项目。把招商引资与结构调整、资源开发、扩大就业、城镇建设和企业改革结合起来，开拓产业招商、配套招商、以商招商的路子，使招商引资在推进工业化、农业产业化和城镇化中产生更大的带动效应。抓好项目库建设，策划包装一批规模大、档次高的项目，打破项目瓶颈。加强对招商引资项目的跟踪落实，抓好项目的履约率、资金到位率和投产率，提高招商引资的质量和水平。进一步落实招商引资领导责任制，强化招商引资“一把手工程”，完善奖惩机制，形成招商引资的强大合力。

把“引进来”和“走出去”作为扩大开放、加快发展的重要举措来抓。在更大范围、更广领域和更高层次上参与国内外经济技术合作和竞争，采取灵活多样的形式，加强宣传造势，全面推介赣州，全方位开放赣州，广交天下朋友，提高赣州在国内外的知名度和影响力。举全市之力，做好2004年世界客属恳亲大会在我市召开的各项准备工作。办好第三届脐橙节。更加积极主动地接轨沿海发达地区，参与国际国内产业组织分工，自觉融入沿海开放型经济圈，促进区域经济优势互补、协调发展，努力使赣州成为沿海发达地区的“三个基地、一个后花园”。

（四）千方百计加大投入，促进经济发展上新台阶

加强以交通为主的基础设施建设。全力配合和抓好赣定高速、赣龙铁路等重大项目建设，确保赣定高速南康至信丰段竣工通车。继续加大市养公路、县乡公路建设力度，全年完成市养公路路基改造和路面各350公里。高标准抓好农村电网和城市电网改造，加快110千伏和220千伏输电网络建设。抓紧杨梅渡大桥及市区与高速公路连接线、梅林大桥、水南大桥、赣州三水厂、污水处理厂、接待中心等市政项目建设，做好“十五”期间开工建设赣韶铁路、西南至东南沿海区域干线加密项目和“县县通”二级公路、500千伏输变电工程的前期准备工作。切实加强工程建设管理，提高工程质量，确保工程顺利实施。

加速以赣州城区为中心的城镇化进程。搞好城市规划，加大城市投入，推进城市建设，加强城市管理，使赣州城区真正成为区域经济发展的中心和城镇化发展的龙头。按照优化布局、扩大规模、提升质量、完善功能的要求，结合工业园区和专业市场建设，加快城镇建设步伐。取消农村人口进入城镇的各种限制，吸引农民进城。大力发展二、三产业，以产业聚集带动人口集聚，以工业园区和专业市场建设推动城镇建设，扩大赣州新城区和以县城为重点的40个中心镇的规模。同时，抓紧农村中心村建设和农房改造。

加大开发区和工业园区建设力度。搞好开发区和工业园区的形态开发、产业开发和功能开发。坚持高起点规划。因地制宜、优化布局，精益求精建设开发区和工业园区。坚持高水平管理。建立符合市场经济规则的管理体制和运作机制，搭建统一、高效、精干的管理平台。坚持高质量服务。加快设施配套和功能提升，引入规范多样的中介服务，健全社会化服务体系，推进开发区和工业园区从单纯依靠政策优势向依靠投资环境综合优势转变，增强开发区和工业园区的吸引力和聚集力。注重塑造开发区和工业园区的特色和差异性，培育特色产业群，打造区域经济板块。

加快投资主体和融资渠道多元化步伐。深化投融资体制改革，运用市场经济手段，对土地资本、产业资本和无形资产等基本要素进行集聚、重组和营运，吸引社会资本、民间资本、市外资金参与各种基础设施建设，争取金融系统加大信贷投入，彻底改变政府单一投资的状况。创新融资方式，鼓励采用资产经营权转让、收费权转让以及股权转让或出售等方式，盘活存量资产，筹集建设资金。完善土地收购储备制度，培育规范土地市场，增加土地收益。积极争取更多的国债资金和专项资金，实现投资主体和融资渠道多元化。

（五）全面实施科教兴市和可持续发展战略，推动各项社会事业上新水平

加快发展科技事业。不断提高企业特别是重点企业的技术创新能力，努力培育拥有自主知识产权、有较强竞争力的高新技术企业和产品。加快科研机构改革步伐，支持科技人员创办、领办、承包科技实体，鼓励科技人员到生产一线进行科技示范活动。扶持发展面向广大中小企业和农户的科技中介服务机构。加强与大专院校和科研单位的联系，逐步形成产、学、研各方积极参与的合作新体制。不断完善人才引进、培养、使用的有效机制。加大科技投入，重奖有突出贡献的人才。

大力发展教育事业。继续高标准普及九年义务教育，不断加大农村义务教育的投入。全面贯彻以县为主的农村义务教育管理体制，积极发展高中阶段教育、职业教育和技工教育。鼓励和发展社会力量办学，推行高中学校后勤服务社会化。进一步做好中小学特别是农村中小学的危房改造和布局调整，改善办学条件。关心和支持驻市大中专院校的改革和发展。全面实施素质教育，着重培养学生的实践能力和创新精神。加强教师队伍建设，实施教师资格制度，努力建设一支高素质的教师队伍。

贯彻基本国策，实现可持续发展。正确处理发展经济与人口、资源、环境的关系，完善与落实计划生育工作责任制，严格控制人口增长，不断提高人口素质。加强土地管理，严格土地审批。加强森林保护和水资源管理，搞好植树造林和退耕还林，防止水土流失。促进矿产资源有效开发利用，防止乱采滥挖。重视生态环境保护，加快污染源治理，制止以牺牲环境、破坏资源为代价的建设，改善城乡环境质量。

切实做好稳定工作。强化安全生产责任制，进一步落实安全生产的各项措施。认真做好民政工作和信访工作。加强社会治安综合治理，大力开展法制宣传教育，深入开展“严打”整治斗争，坚决扫除黑恶势力和“黄赌毒”等社会丑恶现象，打击“法轮功”邪教组织，遏止封建迷信活动，切实维护社会政治稳定。

加强先进文化建设，全面发展各项社会事业。以培养“四有”公民为目标，在文化内容和形式上积极创新，增强先进文化的吸引力和感召力。大力弘扬革命老区精神，加大对革命传统和客家文化等优秀文化遗产的研究开发力度，充分展示赣州文化的魅力。繁荣文艺创作，力创一批有影响力的精品佳作。深入开展群众性精神文明创建和塑造江西人新形象活动，加大社区建设力度，积极争创文明城、卫生城、园林城、优秀旅游城、双拥模范城。认真贯彻落实《公民道德建设实施纲要》，提高全民素质，促进社会主义物质文明、政治文明和精神文明协调发展。加强国防教育，做好民兵、预备役工作。搞好传染病、地方病、职业病防治，巩固和提高农村初级卫生保健成果。广泛开展全民健身活动，提高竞技体育水平。加强统计工作。继续做好民族宗教和外事侨务工作。

三、加强和改进政府工作

在新的一年里，所有政府工作人员都要以贯彻落实党的十六大精神为动力，解放思想，与时俱进，身体力行“三个代表”重要思想，牢记“发展是执政兴国的第一要务”，“本质是执政为民”的执政宗旨。加快政府机构改革，大力推进依法规范行政、科学有序行政、优质高效行政、民主透明行政和清正廉洁行政，创造性地开展政府工作。

进一步更新行政观念。以转变政府职能为核心，理顺政府与企业、政府与市场、政府与社会、政府与公民的关系。切实解决越位、缺位和错位问题，真正把政府职能转到经济调节、市场监管、社会管理和公共服务上来，凡是能由企业为主、市场调节、行业协会和社会中介组织提供服务的领域，政府尽快退出，把主要精力放到抓好事关改革、发展、稳定全局的重大问题上，放到为群众多尽实心、多干实事、多谋实利上。深化行政审批制度改革，进一步削减审批事项，规范审批行为。搞好政务公开，向社会公开政府规章、规范性文件、行政措施和政务活动，提高政府工作的透明度，保障群众的知情权、参与权和监督权，着力塑造政府创优环境、人民创造财富的服务型政府。

进一步转变行政作风。当前各级政府机关仍存在许多不良风气，主要表现为：办事拖拉、推诿扯皮，效率意识不强；思想狭隘、小团体利益作祟，大局意识不强；安于现状、得过且过，进取意识不强；因循守旧、按部就班，创新意识不强等等。这些不良风气，不仅损坏了人民政府的形象，而且引起了人民群众的强烈不满。为此，在政府机关中大力倡导：雷厉风行、说干就干的作风。切实做到能办的事立即办，难办的事想法办，涉及多个部门的事主动协调办，不拖、不等、不推、不靠，减少办事环节，提高工作效率；搞好局部、服从大局的胸襟。政府各部门务必从狭隘的小团体利益中解放出来，坚决克服本位主义、个人主义，勇于牺牲局部利益，不搞上有政策下有对策，以实际行动服务和支持全市工作的大局；敢于作为、争创一流的锐气。有为才有位、有位更有为。面对你追我赶、不进则退的竞争态势，各部门都要自我加压、奋起超越，敢于和善于作为，在江西实施中部地区崛起战略中站前列、当先锋；大胆探索、勇于创新的精神。敢闯敢冒，敢于冲破各种不合时宜的观念、做法和体制，走别人没有走过的路，办别人没有办过的事，以新观念、新举措，破难点、攻难关，坚持实事求是，防止急功近利、虚报浮夸和形式主义。

进一步改进行政方法。适应市场经济体制和加入WTO的要求，从固有的思维定势、行政方式中跳出来。把政府工作转到按市场经济规律办事、与国际惯例接轨、按世贸规则运作上来。减少会议和文件，力戒以会议贯彻会议、以文件落实文件、以讲话指导讲话。做到倾听社情民意在一线，调查研究在一线，真抓实干在一线，解决问题在一线。努力克服命令多、摊派多、指手画脚多的落后行政方法，更多地通过市场之“手”，通过典型带动、示范服务、政策引导来指导经济工作。坚持依法治市，推进依法行政，加大行政执法监督力度，推行行政执法责任制和评议考核制，建立健全行政执法违法和过错责任追究制度。坚持决策的民主化、科学化，问计于群众，求知于实践。自觉接受人大及其常委会的法律监督和人民政协的民主监督。

进一步提高队伍素质。各级政府工作人员要顺应时代前进潮流，坚持自觉学习、终身学习，不断加强理论武装、知识更新和能力培养，力求用世界眼光、国际视野、战略思维指导工作，不断增强工作的前瞻性和预见性。加快形成体现公平竞争、充满生机活力的公务员使用、管理和奖惩机制，激励公务员奋发向上、爱岗敬业、乐于奉献。加强市场经济知识、世贸组织规则、法律知识、管理和科技知识的学习，不断提高驾驭全局的能力、依法行政的能力、科学决策的能力。牢固树立艰苦奋斗的思想，磨练艰苦奋斗的意志，自觉在艰苦奋斗的实践中锻炼和提高自己。加强廉洁自律，完善监督、约束、防范机制，注重从源头上预防和遏止腐败。落实廉政建设责任制，保持公务员队伍的纯洁，塑造政府的良好形象。

各位代表，我们的事业大有可为，赣州的未来充满希望。让我们紧密团结在以胡锦涛同志为总书记的党中央周围，高举邓小平理论伟大旗帜，全面贯彻“三个代表”重要思想，解放思想，与时俱进，自强不息，励精图治，为全面完成今年各项任务而努力奋斗！
